# Foam stability of temperature-resistant hydrophobic silica particles in porous media

**DOI:** 10.3389/fchem.2022.960067

**Published:** 2022-08-25

**Authors:** Sanyuan Qiao, Haibin Yu, Yongan Wang, Lifeng Zhan, Qingwang Liu, Zhenzhong Fan, Ao Sun

**Affiliations:** ^1^ Center of Chemistry for Frontier Technologies, Zhejiang University, Hangzhou, China; ^2^ Key Laboratory of Improving Oil Recovery by Ministry of Education, Northeast Petroleum University, Daqing, China; ^3^ CenerTech Tianjin Chemical Research & Design Institute Co., Ltd, Tianjin, China; ^4^ CNPC, BoHai Drilling Engineering Co, Ltd, Tianjin, China; ^5^ School of Chemical Engineering and Technology, Tianjin University, Tianjin, China

**Keywords:** silane quaternary ammonium salt, hydrophobic silica particle, steam flooding, contact angle, foam stability

## Abstract

The world is rich in heavy oil resources, however, the recovery difficulty and cost are both higher than that of conventional crude oil. To date, the most common method of recovering heavy oil is steam flooding. However, once the steam breaks through the geological formation, gas channeling readily occurs, which leads to a rapid decrease of the steam drive efficiency. To improve the swept volume of steam in the geological formation, a series of hydrophobic silica particles for stabilizing foam was synthesized. This kind of particles used hydrophilic nano silica particles as reactant. Hydrophobic groups with cationic long carbon chains were grafted onto the surface of hydrophilic silica particles by synthetic silane quaternary ammonium salt. When the quantity of silane quaternary ammonium salt used in the modification reaction is different, the product had various degrees of wettability. The hydrophobic particles with the contact angle closest to 90° had the best foam stabilization effect on the betaine zwitterionic surfactant LAB. For LAB solution with mass fraction of 0.3%, the half-life of foam was extended into 160% when the mass fraction of particles was 0.5%. The higher the gas-liquid ratio, the better the plugging effect of foam agent with hydrophobic particles presented in porous media. The adsorption test of hydrophobic particles indicated that hydrophobic particles improved the stability of foam liquid membrane by improving the adsorption capacity of surfactant molecules. The thermal stability of hydrophobic silica particles exceeded 200°C, and the good foam stability made it a potential additive for foam oil displacement in high-temperature geological formation.

## 1 Introduction

Since the end of 2021, the price of conventional energy has been increasing; and the prices of coal, oil, and natural gas have remained high. Fossil energy remains irreplaceable in modern society. Because the price of crude oil continues to increase, heavy oil reservoirs have become important resources in oilfield development. However, it is much more difficult to recover heavy oil than light oil because heavy oil has a high density and poor mobility in geological formations (Speight, 2009; Alvarado and Manrique, 2010). Thermal viscosity reduction is the main technical means of improving the mobility of heavy oil in geological formations. The main methods of thermal recovery include hot water flooding, steam flooding, and steam soaking; steam flooding can substantially heat a geological formation and has the same effects as hot water flooding (Wang et al., 2016). Steam flooding is the most common procedure applied to recover heavy oil blocks.

Whether by steam flooding or steam soaking, the mechanism of enhanced heavy oil recovery is injection of high-temperature steam through a water injection well to heat the geological formation, to improve the seepage capacity of heavy oil in porous media. However, once the steam breaks through the geological formation, gas channeling readily occurs, which leads to a rapid decrease of the steam flooding efficiency (Yang et al., 2016) ^.^ The geological formation in which gas channeling occurs must be blocked in time to expand the swept volume of steam in the formation. Steam foam composite flooding (SFCF) technology is aimed at steam channeling in steam flooding: injection of a foaming agent can block the steam channeling path, improve the steam sweep coefficient, and improve the reservoir development (Pang et al., 2015).

The first SFCF field test was carried out in 1982. Shell conducted 15 months of testing at the Kern River oil field in California, United States. Production reached 4,000 tons, and led to the first report on applying SFCF technology (Dilgren et al., 1982). Since then, SFCF has been an active area of research in the context of elucidating the influence of specific process parameters (Bagheri, 2017; Pang et al., 2018). In 2006, Cao et al. tested the application of nitrogen foam profile control to the Shengli Oilfield—research aimed at the problem of gas channeling of heavy oil blocks, which corresponds to reduced performance of steam soaking in high-water-content wells. The experiment optimized the gas:liquid ratio of the nitrogen foam profile control system at 25°C and 3 MPa; when the gas:liquid ratio was 2:1, the nitrogen foam improved the steam displacement efficiency (Cao et al., 2012). The improvement enhanced the recovery from the high-permeability layer, facilitated entry of steam into the low-permeability layer, and enhanced oil recovery from the low-permeability layer. Therefore, the key factor that affects the foam-plugging steam during SFCF is the stability of the foam in the geological formation. The foam system must be highly stable to plug geological formations (Chen et al., 2021), and must exhibit surface activity for washing oil after the foam fracture (Petkova et al., 2021), to maximize the synergy of the foam and steam over the course of flooding (Zhao et al., 2022).

Since 1907, researchers observed that tiny solid particles could stable emulsion or foam system. With the improvement of characterization technology and the rapid development of micro observation technology, nano materials concerned researchers’ attention in several fields such as food, environment, pharmacy, biology, energy (Chen et al., 2022; Wu et al., 2022; Xie et al., 2022). A foam system that incorporates inorganic nanoparticles (e.g., silica) can exhibit excellent stability when the particles are compatible with the foam agent, such as in terms of the nanoparticle size, wettability, or concentration (Amirsadat et al., 2017). Upon dispersion in the surfactant solution, the nanoparticles distribute at the gas–liquid interface by adsorption. Particles on the interface impart rigidity in a manner that supports the foam, and prevents bubble coalescences and dismutation; and in so doing stabilize the foam (Lesov et al., 2017). These particles can stable the foam system in complex geological formations, unlike other solid particle additives that might damage the channels (Wang et al., 2022). The size of silica nanoparticles is far less than the pore throat radius; thus, when the bubbles fracture, the particles de-attach from the bubble surface and flow directly outward from the geological formation after subsequence display (Guo et al., 2022). Therefore, a foam system that incorporates inorganic nanoparticles causes less damage to a geological formation than other types of foam (Kumar and Mandal, 2017).

Vatanparast et al. (2016) studied the change of dynamic interfacial tension when different dosage of hydrophilic nano silica particles added to the system of low concentration CTAB solution and n-heptane. The experimental results indicated that there was almost no active adsorption of hydrophilic nano silica particles on the oil-water interface, while in the system with surfactant, the particles adsorbed the hydrophilic end of CTAB through the hydroxyl on the surface, so that the hydrophobic chain on the particle surface adsorbed to the oil-water interface. Lesov et al. (2017) studied the stability mechanism of foam formed by tetradecyl trimethylammonium bromide (TTAB) and nano silica particles. The results also found that hydrophilic nano silica particles could not be adsorbed to the gas-liquid interface in the system without surfactant, and can only exist in the bulk phase. After surfactant was added into solution, the disproportionation rate between foam liquid membranes decreased significantly, and a relatively clear particle skeleton observed after foam drying (He et al., 2022), indicated that the necessary condition for particles adsorbed to the gas-liquid interface was hydrophobic surface. Binks (Binks and Murakami, 2006; Binks et al., 2007a; Binks et al., 2007b) et al. also studied the mechanism of environmental response particles in the interface stability. Only when the surface hydrophobicity of nano particles is medium, it could produce a marked effect in the stability of the interface.

The research on foam stabilization of silica particles mostly focused on the interaction between cationic surfactants and hydrophilic particles (Hurtado et al., 2018; Ren et al., 2022), but in the application of foam oil displacement in oil fields, due to the existence of bentonite in the geological formation (Zhao et al., 2020), the application of cationic surfactants was limited.

In this study, the aim was to stabilize foam of surfactants with negatively charged groups, and synthesize hydrophobic silica nanoparticles with cationic hydrophobic groups through silica oxygen bonds. The particles were characterized, and the stability of the particles (which exhibited various degrees of wettability with betaine surfactant) was studied. The foam-stabilized particles in this study are pertinent to onsite application of steam foam composite flooding technology.

## 2 Materials and methods

### 2.1 Materials

Silica particles of various degrees of wettability were synthesized. Tetraethyl orthosilicate, ammonia, lauric acid, ethanol (99.8%) (3-chloropropyl)trimethoxysilane, and 3-dimethyl aminopropyl amine were purchased from Aladdin, Shanghai, China; and used as received without further purification. The surfactant lauroylamide propylbetaine (LAB; purity = 35%) was purchased from Guangdong Wengjiang Chemical Reagent, Shaoguan, China.

### 2.2 Synthesis and characterization of silica particles

#### 2.2.1 Synthesis of silica particles of various degrees of wettability

First, 8 ml of absolute ethanol, 4.5 ml of ammonia, and 12 ml of distilled water were added to a flask in various proportions; and stirred at 350 r/min, 25°C. Then 2.25 ml of tetraethyl orthosilicate was dissolved in 22.25 ml of ethanol, mixed, and quickly poured into the flask. The solution was stirred at 1,200 r/min for 1 min, the speed was reduced to 350 r/min, and the reaction was continued at 25°C for 2 h. Upon completion of the reaction, the solution was centrifuged at 12,000 r/min for 15 min, the supernatant was removed, and the particles were washed with distilled water; this procedure was repeated 5×. The silica particles were vacuum-dried at 80°C overnight.

**FIGURE 1:**
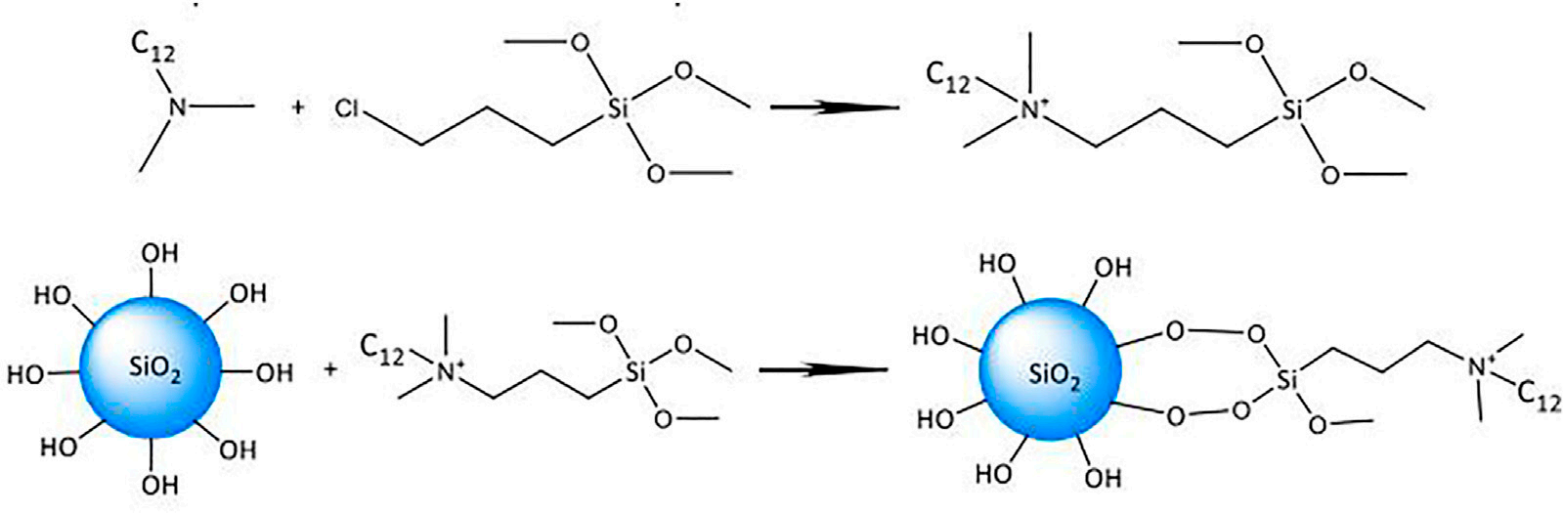
Synthesis and modification of functional silica particles.

**FIGURE 2:**
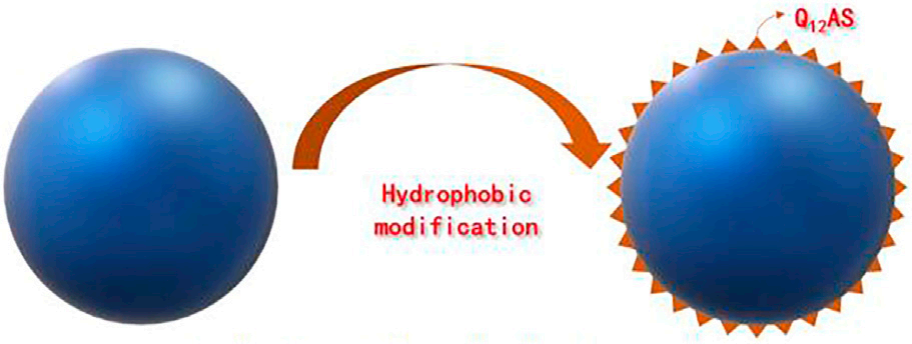
Surface modification of silica particles.

The silica particle modification was performed by the procedures described in our previous work (Sun et al., 2019). As shown in Figure 1, the silica particle modifier, a silane quaternary ammonium salt (Q_12_AS), was synthesized with lauric acid and (3-chloropropyl)trimethoxysilane. Silica particles (0.5 g) were ultrasonically dispersed in 50 ml of ethanol for 15 min; then a certain quantity of Q_12_AS (either 0.5, 1.0, or 2.0 g) was added, under stirring at 50°C for 6 h. Upon completion of the reaction, the solution was centrifuged at 12,000 r/min for 15 min, the supernatant was removed, and the particles were washed with ethanol; this procedure was repeated 5×. The modified silica particles were vacuum-dried at 80°C overnight. The surface modification of silica particles is shown in Figure 2.

#### 2.2.2 Wettability evaluation and microscale morphology of silica particles

The wettability of the silica particles was tested by measuring the contact angle of a water drop. First, a glass slide was coated with the particles. Then 0.05 g of various degrees of wettability of silica particles were ultrasonically dispersed in 10 ml of ethanol for 15 min. Ten microliters of solution were deposited onto the glass slides with a microinjector. The glass slides were vacuum-dried at 50°C for 4 h, and then the contact angle of water droplets on the slides was measured. The microscale morphology of the silica particles was observed with a FEI quanta 450FEG field-emission scanning electron microscope, and a JEOL-JEM2100 transmission electron microscope. The samples were dispersed with absolute ethanol as the solvent prior to imaging.

#### 2.2.3 Adsorption capacity of hydrophobic silica particles

The adsorption capacity of silica particles and the effect of the silica particles on the surface tension of various ionic surfactant solutions were tested by surface tension experiments. The change in the surface tension of surfactants was measured at various concentrations by adding nanoparticles. A surfactant solution 0.1 mol/L was prepared with distilled water, then the solution was separately diluted to 1.0 × 10^–2^ mol/L, 1.0 × 10^–3^ mol/L, 1.0 × 10^–4^ mol/L and 1.0 × 10^–5^ mol/L. A determined quantity (mass percent) 0.5% of silica particles were ultrasonically dispersed into the surfactant solution. Then the surface tension of the solution was tested at room temperature.

#### 2.2.4 Foam stability of silica particles

The foam stability of the silica particles was evaluated by the Waring–Blend method. LAB was used as a foam agent. A surfactant solution (mass percent: 0.3%) was prepared with distilled water. The silica particles were ultrasonically dispersed in the surfactant solution. Then the mixture was stirred at 6,000 r/min for 60 s. Next, the foam was transferred into a measuring cylinder and the time required for 50 ml of liquid separation was recorded to determine the stability of the foam. The microscale morphology of the foam was observed with an inverted microscope.

The plugging ability of the foam system in porous media was studied by core experiments. Water and air were injected into a 60-mm-long, 25-mm-diameter artificial core at various gas:liquid ratios. The change of the pressure at both ends of the core was recorded upon reaching stable values. The plugging performance of the foam system in porous media was determined by comparing the differential pressure of the core before and after injecting the foam system.

**FIGURE 3:**
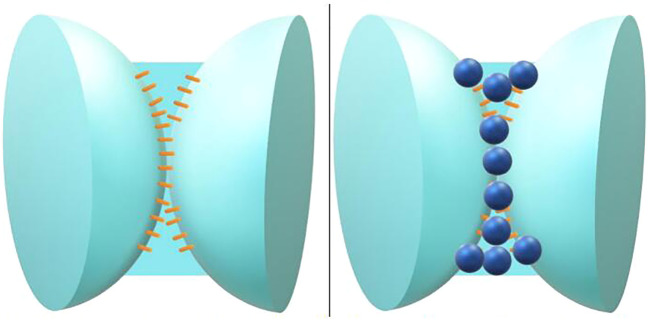
Surfactant stabilized foam gas-liquid interface (left figure), hydrophilic silica particles are distributed at the gas-liquid interface (right figure).

**FIGURE 4:**
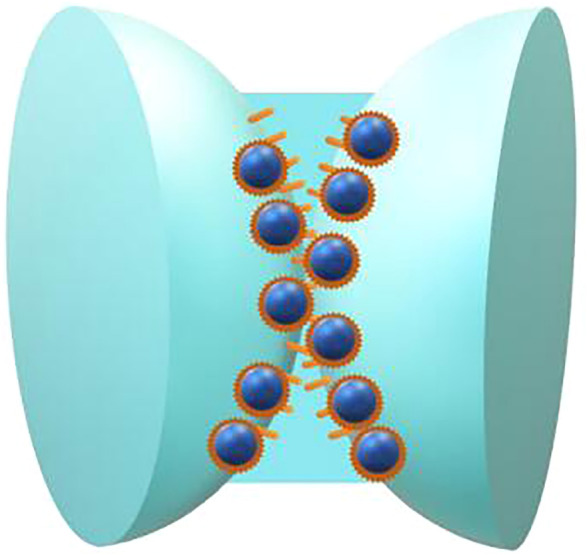
Schematic diagram of hydrophobic particle stabilized foam.

The hydrophilic particle stabilized foam is shown in Figure 3. The hydrophobic silica particles are easier to be adsorbed on the side close to the gas phase at the gas-liquid interface, which increases the strength of the interface facial mask. The hydrophobic particle stabilized foam is shown in Figure 4.

## 3 Results and discussion

### 3.1 Characterization

#### 3.1.1 FT-IR

**FIGURE 5:**
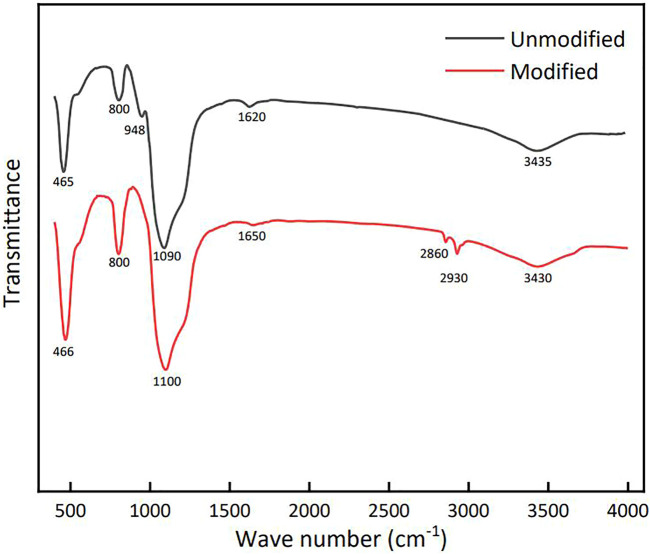
FT-IR spectra of functional SiO2 before and after modification.

The infrared spectrum was measured by Irtracer-100 Fourier transform infrared spectrometer (Shimadzu company, Japan), the obtained spectrogram is shown in Figure 5.

A typical wide peak appeared at 3,435 cm^−1^, belonging to the oh antisymmetric stretching vibration peak adsorbed on the surface; In addition, 1,620 cm^−1^ is the bending vibration peak of H-O-H in the bonded water on the SiO_2_ surface; The strong absorption peak of 1,090 cm^−1^ is Si-O-Si antisymmetric stretching vibration peak; The Si-O bond symmetric stretching vibration peak appears at 800 cm^−1^ and 465 cm^−1^; It is worth noting that the inconspicuous peak at 948 cm^−1^ is the bending vibration absorption peak of Si-OH, and the -OH group on the surface of hydrophilic silica is the grafting site of modified silica.

The modified particles have methyl and methylene absorption peaks at 2,879 cm^−1^ and 2,948 cm^−1^; The hydroxyl bending vibration absorption peak at 960 cm^−1^ disappeared completely, and the hydroxyl bending vibration peak and antisymmetric stretching peak at 1,662 cm^−1^ and 3,455 cm^−1^ weakened; The enhancement of Si-O absorption peaks at 465 cm^−1^ and 800 cm^−1^ indicates that the hydroxyl groups on the surface of silica particles have been grafted into siloxane bonds by siloxane modifiers.

#### 3.1.2 TG/TGA

**FIGURE 6:**
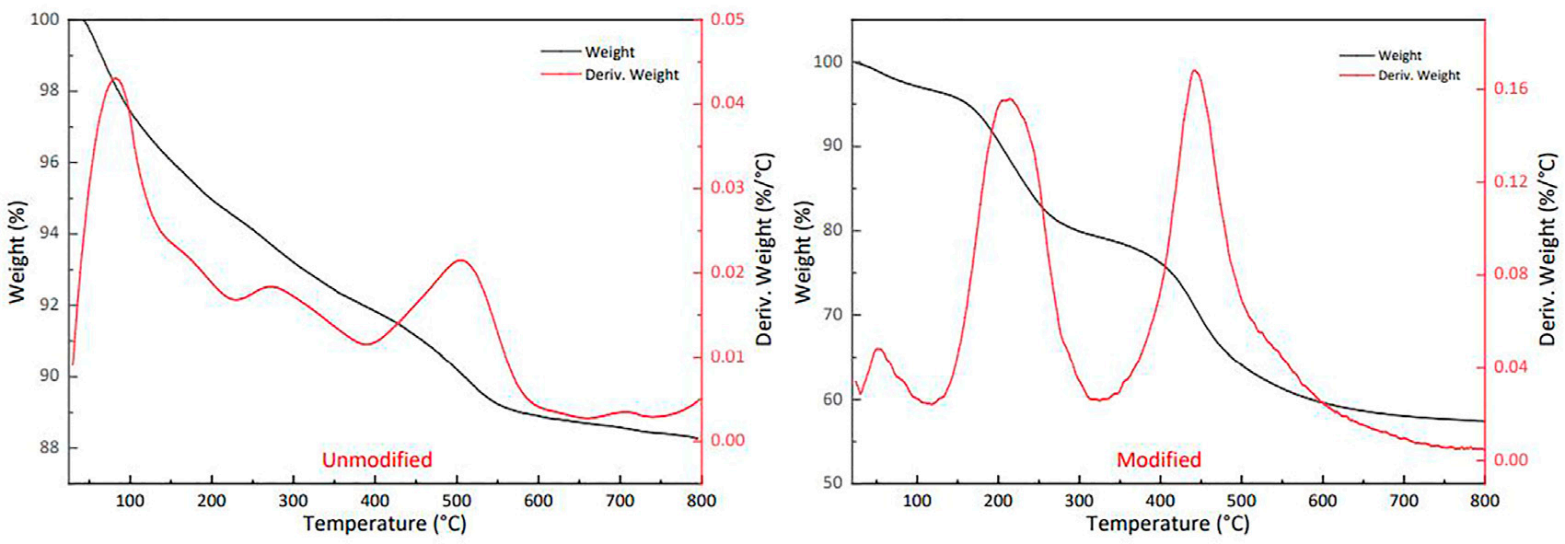
Thermogravimetric comparison curve of SiO_2_ particles before and after modification.

The thermal properties of SiO_2_ particles were detected under a nitrogen atmosphere by thermogravimetric analysis (TGA) on a Q50 analyzer from TA Instruments. The carrier gas flow is 60 ml/min, the heating rate is 10°C/min, and the scanning temperature range is 65–800°C. The results are shown in Figure 6.

The surface of nano silica particles synthesized by solution method has a large number of hydroxyl groups. Through thermogravimetric analysis, it can be seen that the hydrophilic silica particles dried in vacuum appear the first weight loss peak when the temperature is heated to 100°C, which is the weight loss peak produced by the evaporation of water adsorbed on the surface of hydrophilic particles; The second small weight loss peak appeared near 280°C, which should be the weight loss peak produced by the bound water of hydroxyl groups on the surface of particles; The weight loss peak at 500°C corresponds to the dehydroxylation weight loss peak caused by the breaking of silicon oxygen bond in nanoparticles.

After modification, the thermogravimetric curve of silica changed significantly. First, the weight loss peak of adsorbed water disappeared at 100°C, indicating that the surface hydroxyl of silica particles lost hydrophilicity after grafting hydrophobic groups through modifiers; The weight loss peak near 200°C was caused by the breaking of C-N bond and C-C bond in the hydrophobic group on the particle surface; Another weight loss peak at 450°C is Si-O bond breaking, which leads to the decomposition of modifier on the particle surface. Through thermogravimetric analysis, it can be seen that the modifier can effectively shield the hydrophilic groups on the surface of the particles, so that the particles are hydrophobic, and the hydrophobic particles have good thermal stability through the grafting modification of Si-O bond. The hydrophobic particles are completely unaffected under the environment below 200°C, and are also in a metastable state at 200–450°C: Through the partial decomposition of the modified group grafted by Si-O bond, the particle surface is still hydrophobic, and the particle surface will not be reduced to hydrophilic state until it exceeds 450°C.

The thermal stability test also showed that the structure of the modified particles began to damage at 200°C, and the surface decomposition began at 450°C, indicating that the working conditions of this material below 200°C were stable.

### 3.2 Wettability of silica particles

**FIGURE 7:**
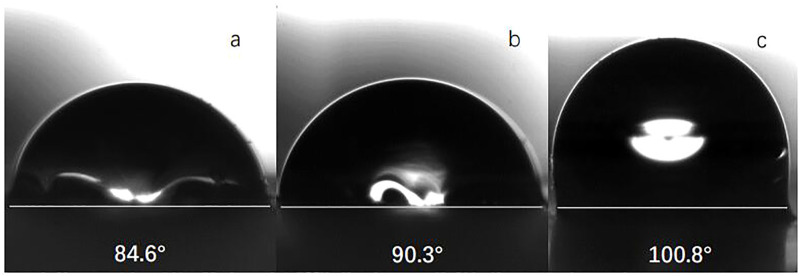
Image of static water contact angle of nano silica particles coating modified by different modifiers dosage [**(A)**: 1:1; **(B)** 2:1; **(C)** 4:1]

Figure 7 shows the water contact angle of silica particles with various degrees of surface wettability. With increasing modifier dosage, the contact angle of the silica particles increased; when the modifier dosage was 4× that of the silica particles by mass, the contact angle was 100.8°. There are many exposed hydroxyl groups on the surface of bare silica particles; the modifier was grafted onto the particles by Si-O bonds. Presumably, steric effects induced by the long carbon chains of the modifier precluded the solvent from accessing the exposed hydroxyl groups on the nanoparticle surface, such that the surface wettability of the particles changed from hydrophilic to hydrophobic. One can use exposed hydroxyl groups as grafting sites for the silane modifier. Thus, when the quantity of modifier was increased, the coverage of the silane modifier increased, and the hydrophobicity of the particles increased.

**FIGURE 8:**
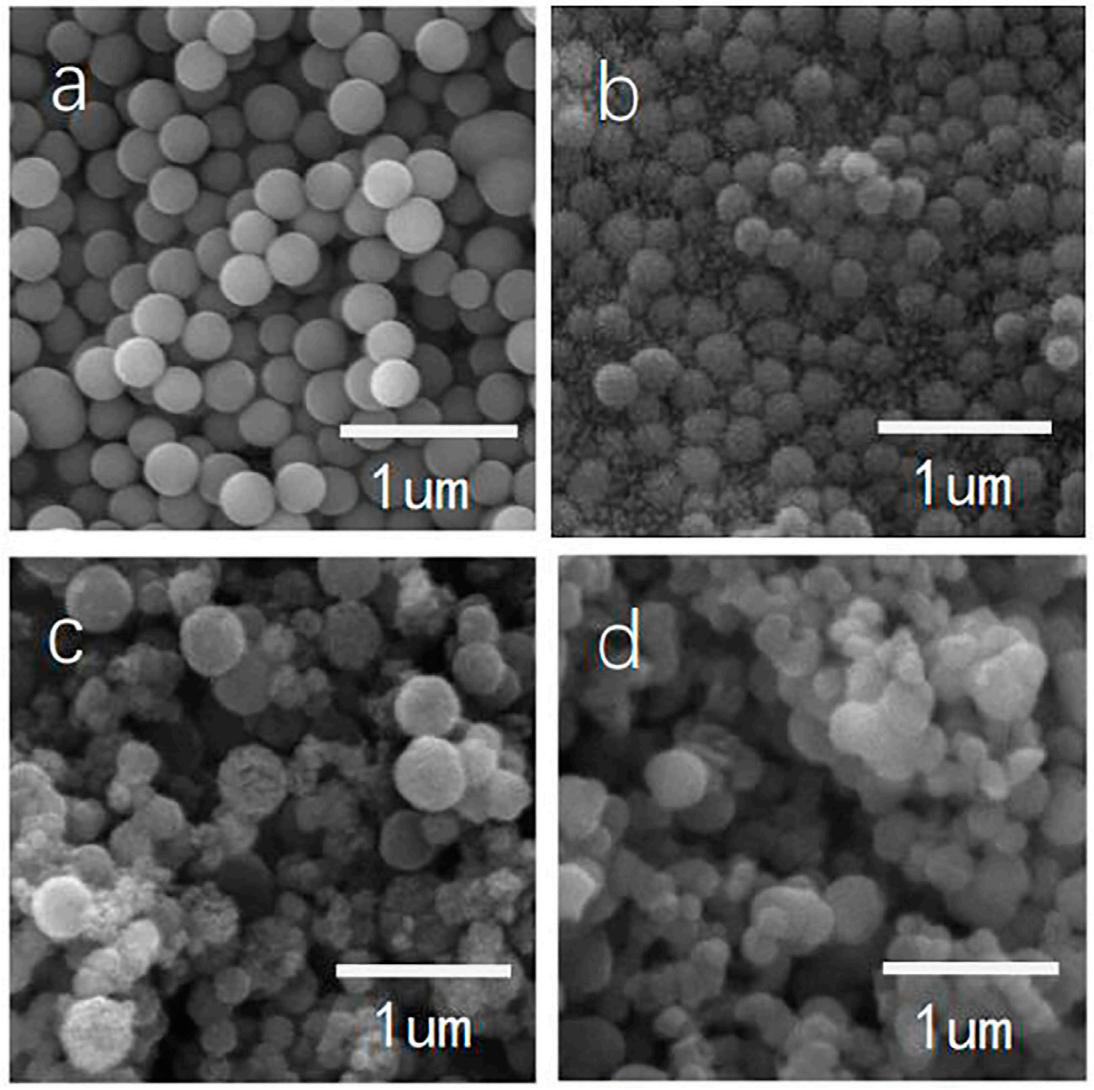
Scanning electron microscopy images of bare and modified nanoscale silica particles.

The microscale morphology of the silica particles indicated differences between bare and modified particles. Figure 8 shows scanning electron microscopy images of various silica particles. The surface of the particles changed with increasing modifier dosage. The bare silica particles had a spherical shape, compact structure, and smooth edges (Figure 8A). The modified silica particles had irregular bulges on the surface, and the roughness of the surface increased with increasing modifier dosage (Figures 8B,C). When the modifier dosage was increased to 4× the particle mass, there were obvious bulges on the particle surface and irregular structures distributed among the particles (Figure 8D). The excessive local hydrolysis rate, attributable to the increased modifier concentration, was the reason that these irregular structures formed (comprised of amorphous silica).

**FIGURE 9:**
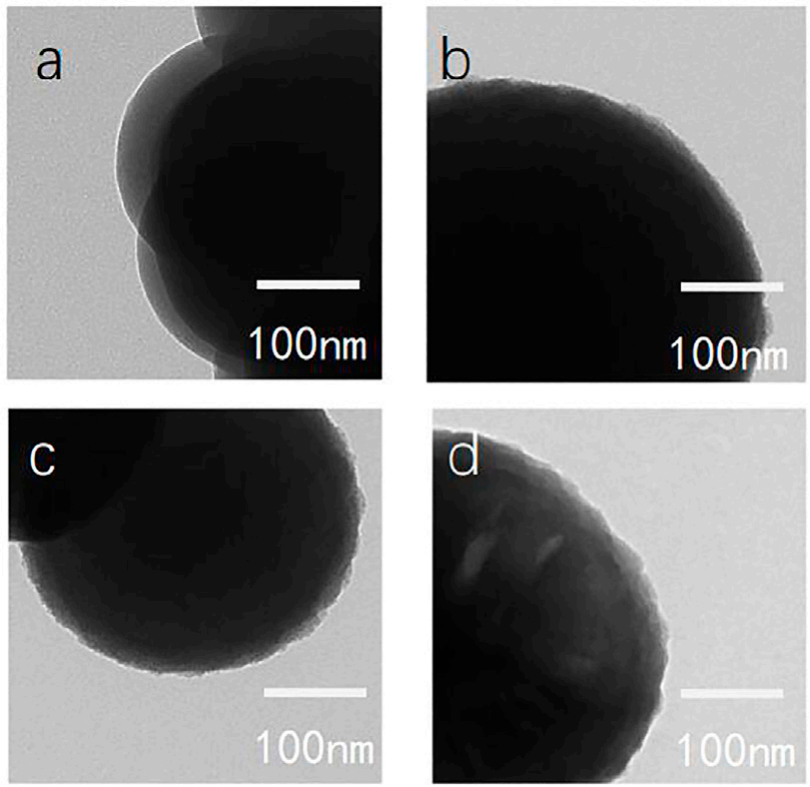
Transmission electron microscopy images of bare and modified nanoscale silica particles [**(A)**: bare SiO_2_; **(B)** C_12_QAS 1:1 SiO_2_; **(C)** C_12_QAS 2:1 SiO_2_; and **(D)** C_12_QAS 4:1 SiO_2_].

Figure 9 shows transmission electron microscopy images of various silica particles dispersed in ethanol. The morphology of the grafts on the particle surface was more evident than by scanning electron microscopy. The edge of the particles changed with increasing modifier dosage (Figures 9A–D). When the dosage of the modifier was small, the edges of the particles had villous-like bulges, which gradually changed to remarkable in path with increasing modifier dosage.

### 3.3 Adsorption capacity of silica particles to LAB

**FIGURE 10:**
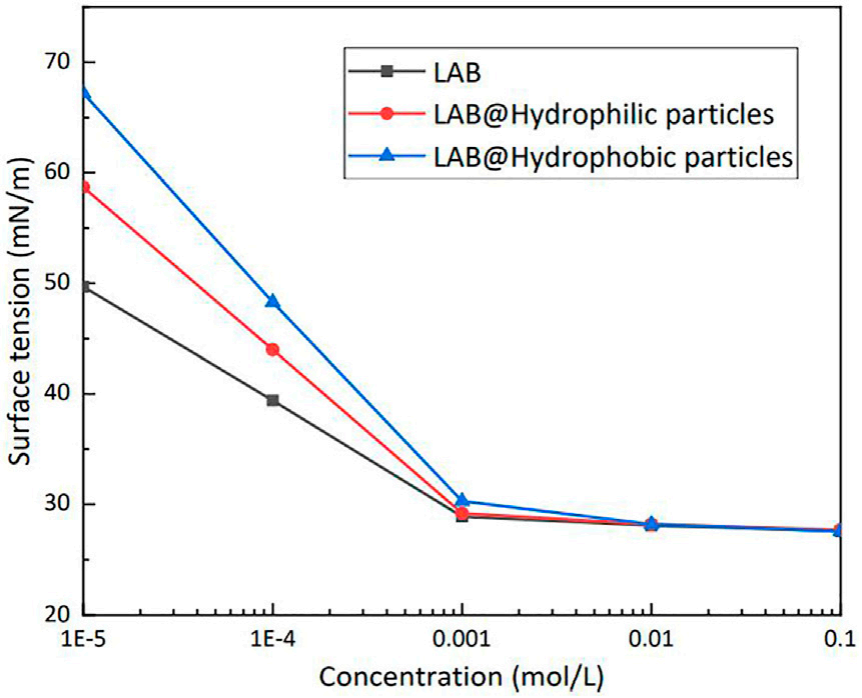
Effect of particles on the surface tension in lauroylamide propylbetaine (LAB) solutions of various concentrations (25°C).

Figure 10 shows the surface tension of LAB solutions at various concentrations. The silica particle mass percent was 0.5%. When the LAB concentration was <1 × 10^–3^ mol/L, the surface tension of the solutions that contained particles increased substantially, which was caused by the adsorption of surfactant molecules onto the surface of the silica particles. Hydrophobic particles can adsorb more surfactant molecules than hydrophilic particles, such that at the same concentration a solution that contains hydrophobic particles has a higher surface tension than a solution that contains hydrophilic particles. With increasing LAB concentration, the surface tension of the mixture solutions approached. When the concentration of the solution exceeded the critical micelle concentration, adding particles did not further impact the surface tension of the solution.

### 3.4 Effect of silica particles on the stability of foam

**FIGURE 11:**
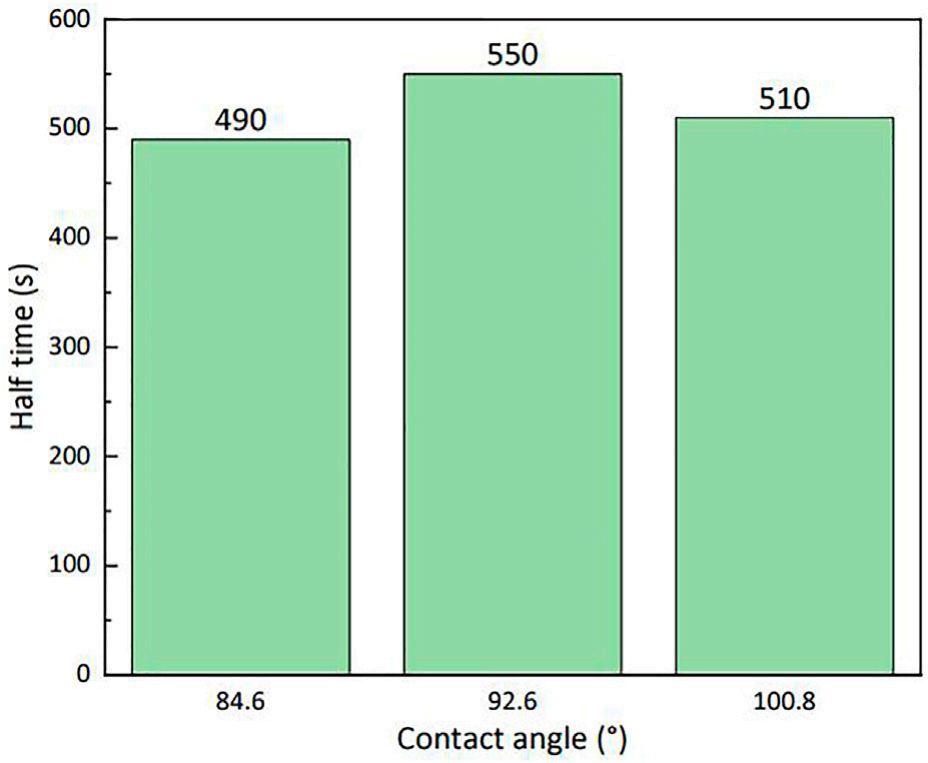
Stability of lauroylamide propylbetaine foam with silica particles of various degrees of wettability (25°C).

Particles of different wettability had different stabilities to the foam formed by the LAB solution (Figure 11); the stability of the particles with a contact angle close to 90° can be improved by 60 s compared with that of the least-stable particles. Thus, particles with contact angles close to 90° were selected to evaluate the stability of the foam at various particle mass percentages in 0.3% (by mass) LAB.

**FIGURE 12:**
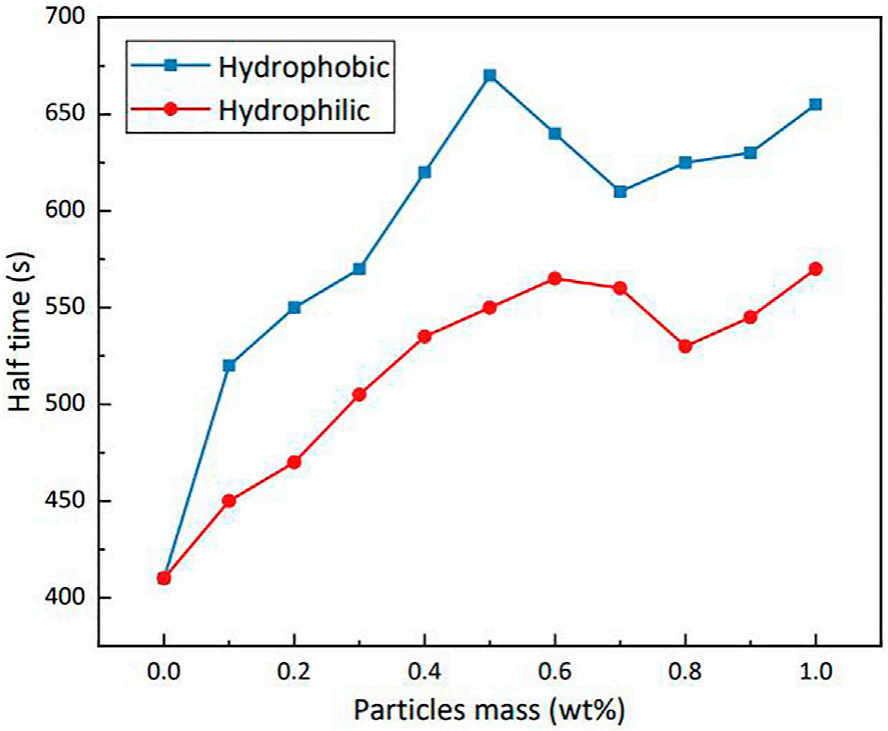
Stability of LAB foam at various silica particle dosages (25°C).

Figure 12 shows the effect of silica particles (with contact angles close to 90°) and hydrophilic particles on the stability of LAB foam at various particle mass percentages. Compared with hydrophilic particles, the modified hydrophobic particles had better foam stability, and adding particles substantially improved the stability of the foam when the particle dosage was <0.5% (by mass). The half-life of foam formed by the LAB solution without particles was 420 s; when adding 0.5% (by mass) hydrophobic particles, the half-life reached 670 s, an increase of 160%. However, when the particle dosage exceeded 0.5% (by mass), the stability of foam did not improve; the stability even slightly decreased.

### 3.5 Stability of foam in porous media

**FIGURE 13:**
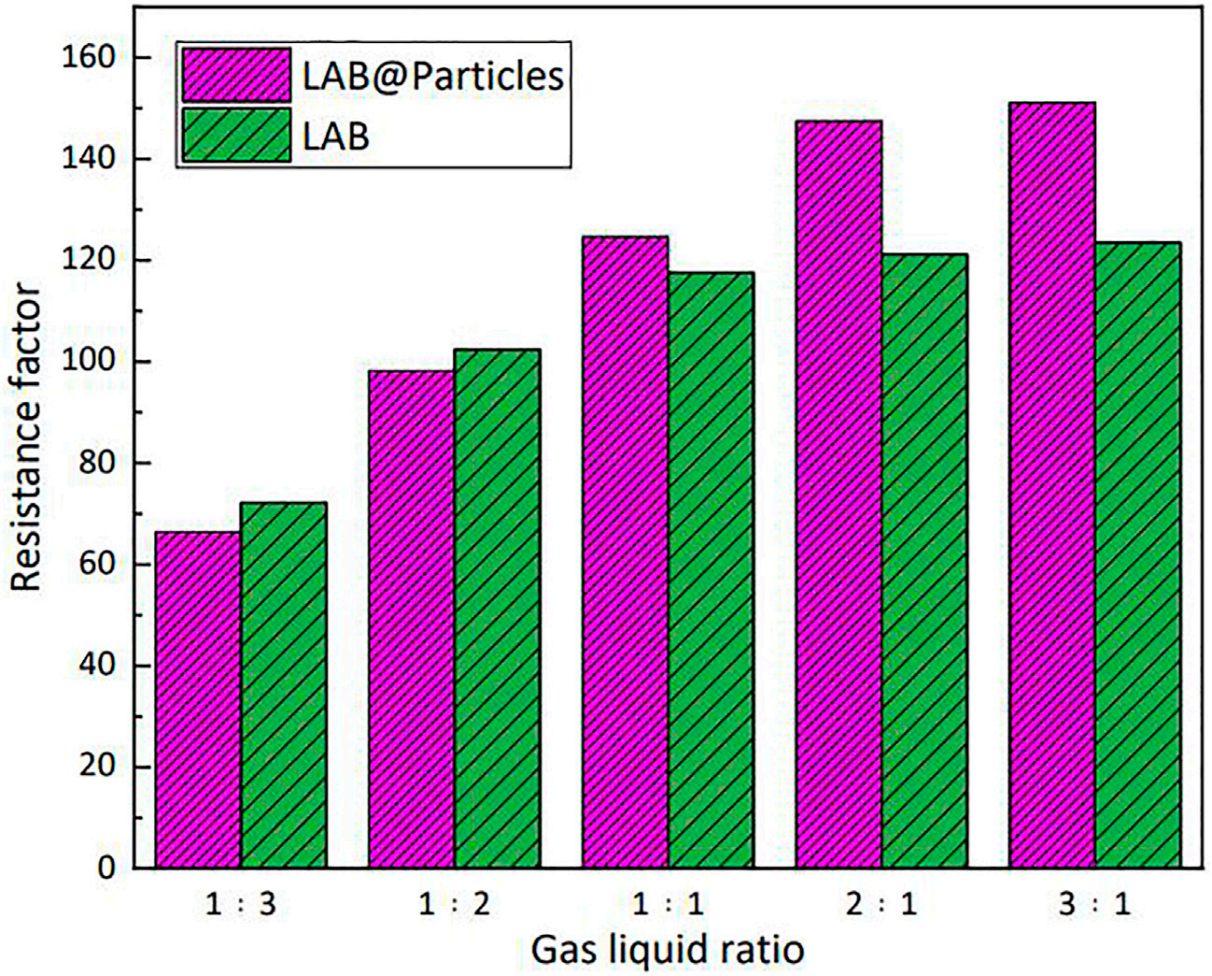
Silica particles enhanced the plugging ability of lauroylamide propylbetaine (LAB) solution in porous media.

The plugging ability of the foam system in porous media is attributable to the fluid lock effect. In porous media, one can achieve selective plugging in steam flooding because oil corresponds to decreased stability of the foam. The seepage resistance of high-temperature steam increases in high-permeability geological formations and is diverted to low-permeability geological formations with high oil content, in a manner that improves the steam sweep volume. Figure 13 shows the plugging effect of hydrophobic nanoparticles in porous media. When the gas:liquid ratio was low, the plugging ability of the nanoparticles to surfactant solution in porous media was minimal. However, with increasing gas:liquid ratio, the plugging effect of silica particles on the foam agent in porous media was substantially improved. When hydrophobic silica particles were added to the foam system, the surface tension of the solution was slightly increased because of the strong adsorption capacity of the particle surface to the surfactant. Therefore, in porous media, the surface of the liquid film can absorb more surfactant molecules, in a manner that retains the stability of the liquid film.

### 3.6 Analysis of foam stabilization mechanism of silica particles

The stability of solid particles to foam depends on the particles’ adsorption to the liquid film, in accordance with the following equation for calculating the desorption energy of particles at an interface (proposed by Binks) (Binks and Lumsdon, 2000):

$$\Delta G_{remove}=\pi R^{2}\gamma_{OW/AM}{\left(1\pm \cos\theta \right)}^{2}$$ (1)

where *R* is the particle radius, *γ* is the ratio of the surface tension between the air and liquid, and *θ* is the contact angle of the particles at the interface. The term in parentheses represents the vector of the motion of the silica particles. When the particles move from the aqueous phase to the interface, the vector motion is positive; otherwise, it is negative. At a contact angle *θ* of 90°, the desorption energy ∆*G* is at a maximum. Thus, when the wetting degree of the particles is close to 90°, the desorption energy at the gas–liquid interface is large—which can enhance the stability of the foam.

**FIGURE 14:**
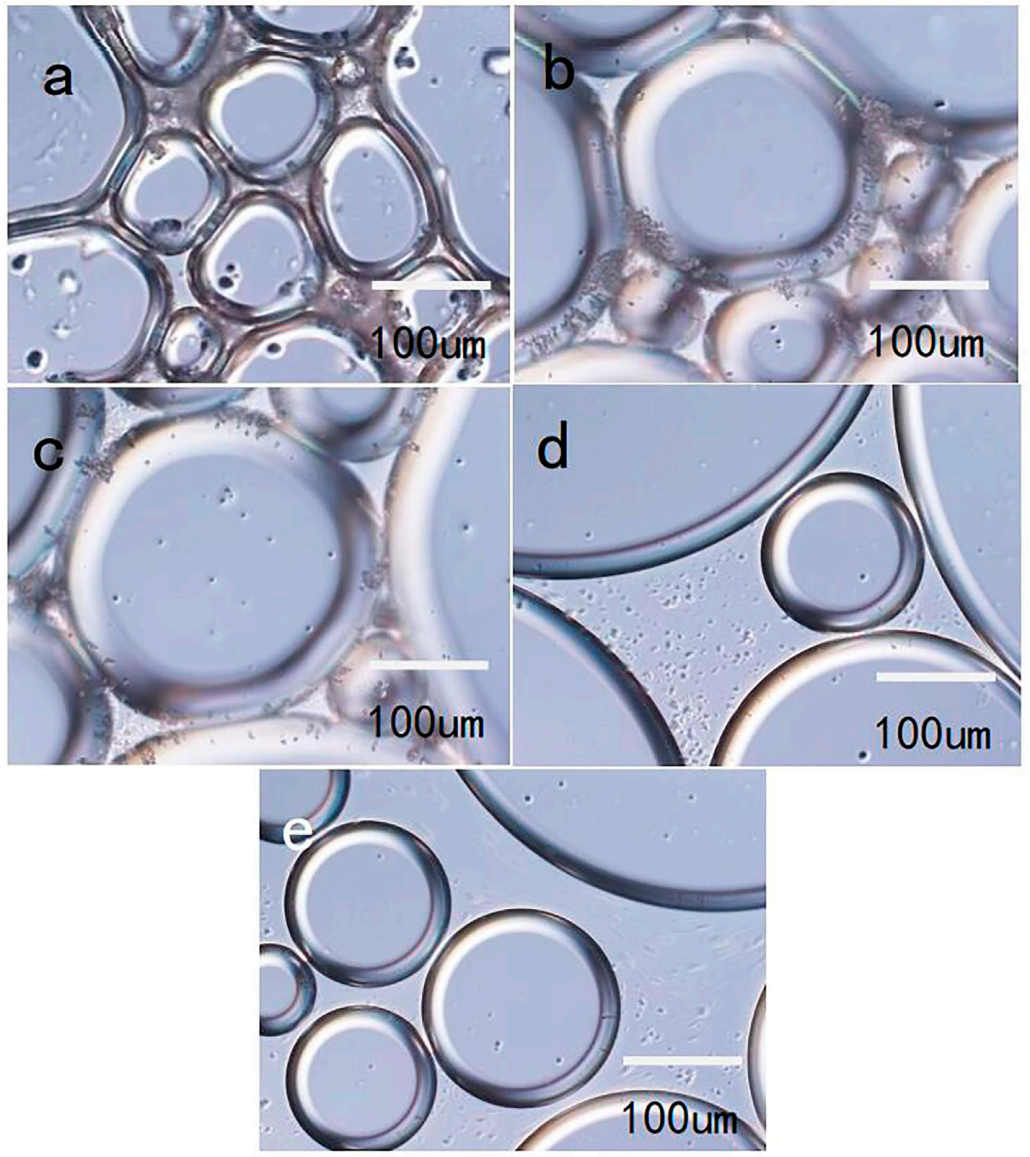
Foam morphology after various particle additions (**(A)**: 1 wt%; **(B)** 0.7 wt%; **(C)** 0.5 wt%; **(D)** 0.3 wt%; and **(E)** 0.1 wt%).

Figure 14 shows images of the foam formed by LAB solutions and particles of various mass percentages, as observed with an inverted microscope. In accordance with the foam morphology at various silica particle dosages, when the particle dosage was 1.0 wt%, there was particle agglomeration in the liquid phase (Figure 14A). Thus, agglomeration of particles in the liquid phase can decrease drainage of the foam to a certain extent; but the particles are also affected by gravity sedimentation, which decreases the stability of the foam.

When the dosage of silica particles was 0.7% (by mass), aggregates of particles were concentrated on the liquid film, whereas the quantity of aggregates in the liquid phase was reduced (Figure 14B). This leads to tensile stress of the particle aggregates on the liquid film under the action of gravity; the aggregates cannot form a blocking network structure in the liquid phase. Therefore, the foam stability performance will be less than that of a foam system that forms in the bulk phase. When the particle dosage was 0.5% (by mass), the particles were dispersed relatively evenly on the liquid film, and the quantity and scale of aggregates were substantially smaller than when the particle dosage was larger (Figure 14C). The coverage of particles on the liquid film was high, the mechanical strength of the liquid film was high, and the effect of gravity was low; such that the foam can be stable for a relatively long time.

Figure 14D shows an image of the foam when the particle dosage was 0.3% (by mass). The particles were distributed at the interface and in the bulk phase, but there were no obvious aggregates; indicating that during foaming, the energy was insufficient to disperse all of the particles from the bulk phase to the interface (Figure 14E), which is why the stability of the foam improved by only a small extent when the particle dosage was low.

In accordance with the results of previous foam-plugging experiments in porous media, adsorption of nanoparticles to the gas–liquid interface during foaming uses energy, which can also explain the fact that when the gas:liquid ratio was low, the presence of nanoparticles slightly reduced the plugging ability. When the gas:liquid ratio was increased, the foaming ability of the system in porous media was improved, and the stabilizing effect of the nanoparticles on the foam was maximized.

## 4 Conclusion

The present study used 3-chloropropyl trimethoxysilane as a quaternary modifier of hydrophilic silica particles. Hydrophobic silica particles with various degrees of wettability were synthesized by controlling the quantity of silane quaternary ammonium salt. The stability imparted by various particles to foam was evaluated by the Waring–Blend method. The particle contact angle was close to 90° and the half-life of the LAB solution was increased by 140 s. The effects of various particle dosages on the stability of LAB solution were evaluated. At a concentration of 0.3% (by mass) LAB, the optimal stable state was achieved when the particle dosage was 0.5% (by mass). The stability of the foam after adding hydrophobic particles was extended by 160% compared with that of the foam without particles. The synthesized hydrophobic silica particles also improved the plugging performance of LAB solution in porous media. When the gas:liquid ratio reached ≥2:1, the plugging capacity was improved by >20%. The test results of the microstructure of the foam and the surface tension of the solution containing particles indicate that the silica particles modified with a silane quaternary ammonium salt had a stronger adsorption capacity for LAB, and improved the stability of the foam formed by LAB solution.

## Data Availability

The raw data supporting the conclusion of this article will be made available by the authors, without undue reservation.

## Nomenclature

SFCF: Steam foam composite flooding
Q_12_AS: The silica particle modifier, a silane quaternary ammonium salt (Q12AS), was synthesized with lauric acid and (3-chloropropyl) trimethoxysilane.
LAB: Lauramidopropyl betaine (N,N-Dimethyl-N-dodecanoylaminopropylbetaine)
